# Clinical characteristics and outcomes in microscopic polyangiitis patients with renal involvement: a study of 124 Chinese patients

**DOI:** 10.1186/s12882-019-1535-3

**Published:** 2019-09-02

**Authors:** Jia Shi, Qing Shen, Xue-Mei Chen, Xiao-Gang Du

**Affiliations:** 1 Department of Nephrology, The First Affiliated Hospital of Chongqing Medical University, Youyi Road 1, Chongqing, 400042 China; 2 Emergency Department, The First Affiliated Hospital of Chongqing Medical University, Youyi Road 1, Chongqing, 400042 China

**Keywords:** Microscopic polyangiitis, Renal involvement, Clinical characteristics, outcome

## Abstract

**Background:**

Microscopic polyangiitis (MPA) is a systemic autoimmune disease, and renal involvement is frequently present in MPA. MPA patients with renal involvement may have a worse prognosis. In this study, we aimed to evaluate the prognostic factors associated with all-cause death and renal survival in MPA patients with renal involvement.

**Methods:**

A retrospective observational cohort study was performed. One hundred twenty-four patients newly diagnosed with MPA with renal involvement excluding end-stage renal disease (ESRD) who were hospitalized at the First Affiliated Hospital of Chongqing Medical University from January 2012 to July 2017 were included. All the survivors were followed up with until July 2018. The clinical and laboratory data at the time of the initial MPA diagnosis were collected, and the predictive values of the variables for mortality and renal outcome were analysed.

**Results:**

Among the 124 patients, 52 were men (41.9%) and 72 were women (58.1%), and the age range was from 25 to 85 years (63.9±10.6 years). Seventy-six patients (61.3%) had pulmonary involvement. Multivariate Cox analysis revealed that age≥65 years (HR: 2.437; *P*=0.021), serum creatinine≥500 μmol/L (HR=2.207; *P*=0.009) and interstitial lung disease (ILD) (HR=2.366; *P*=0.013) were associated with mortality. Cox multivariate analysis identified that serum creatinine≥500 μmol/L (HR=8.236; *P*<0.001) and ILD (HR=2.649; *P*=0.001) were independent detrimental factors for renal survival, and immunosuppressive treatment was a protective factor for renal survival (HR=0.349; *P*=0.001). The area under the ROC curve (AUC) of the serum creatinine level at diagnosis was 0.705 for mortality and 0.870 for progression to ESRD or the doubling of serum creatinine.

**Conclusions:**

Age, serum creatinine level at diagnosis and ILD were independent predictors of mortality in MPA patients with renal involvement. Serum creatinine level at diagnosis, ILD and immunosuppressive treatment were independently related to renal survival.

## Background

Antineutrophil cytoplasmic antibody (ANCA)-associated vasculitis (AAV) is a systemic autoimmune disease characterized by necrotizing small-vessel inflammation, and it is associated with the presence of myeloperoxidase (MPO)-ANCA or proteinase 3 (PR3)-ANCA. AAV compromises microscopic polyangiitis (MPA), granulomatosis with polyangiitis (GPA) and eosinophilic granulomatosis with polyangiitis (EGPA).

The epidemiology and clinical phenotype of AAV patients are different among geographical regions. MPA is the predominant subtype in China and Japan, whereas GPA is more frequent in Northern Europe [1, 2]. The epidemiological manifestations of AAV can be different even among European populations. Higher incidences of MPA were reported in more southern countries, and lower incidences were reported in more northern countries [1, 3]. The annual incidence of MPA was reported as 2.7 per million in Norway and 11.6 per million in Spain [4]. Regarding the phenotype, MPO is the major target antigen of ANCA in Chinese patients, constituting approximately 80% of AAV patients [5, 6], whereas two-thirds of AAV patients in the United Kingdom were PR3-ANCA-positive [1]. Therefore, the clinical characteristics and outcomes of MPA patients may be different according to different geographic locations. Regarding the ANCA phenotype, a study showed that 67% of MPA patients were MPO-ANCA positive in the United Kingdom [7] and a higher ratio of MPO-ANCA positivity was reported in Spain (90.4%) and Japan (varying from 97.1% to 97.4%) [8–10].

Because GPA and PR3-ANCA positivity is more common in European countries, few European studies have focused mainly on the outcome of MPA or MPO-ANCA-positive patients. MPA is a multiple-system disease, involving primarily the kidneys and lungs, and it is characterized by necrotizing glomerulonephritis and pulmonary capillaritis [11]. Many clinical indexes have been reported to have prognostic value in AAV patients, such as age [6, 12, 13], haemoglobin level [8], albumin level [14], Birmingham Vasculitis Activity Score (BVAS) [15, 16] and comorbidities [17]. Many studies have shown that AAV patients with worse renal function have a poorer prognosis [11, 13, 15, 18], including patients with MPA [14, 19]. Given that almost all MPA patients develop renal involvement during the disease course [4, 20], the relationship between renal function and prognosis deserves to be studied. Among patients with AAV, interstitial lung disease (ILD) is more common in Japan (29-39%) [8, 21] than in European countries [22, 23], and AAV patients with ILD has a worse prognosis. However, few data are available regarding the predictors of MPA patients with renal involvement in the Asian region, including China. Since the introduction of immunosuppressive treatment, the prognosis of MPA patients has significantly improved [24–26]. However, immunosuppressive treatments have many severe side effects, including secondary infection, leukopenia, and secondary infection was shown as the leading cause of death during the first year after diagnosis [6]. Consequently, the risk factors of prognosis should be considered when designing therapeutic regimens, especially when designing active immunosuppressive treatment. Our working hypothesis was that age, hemoglobulin level, albumin level, BVAS, renal function, ILD and immunosuppressive treatment can provide prognostic information for Chinese MPA patients with renal involvement.

In the present study, we retrospectively analysed the clinical characteristics and outcome of 124 MPA patients with renal involvement to identify risk factors for renal prognosis and survival.

## Methods

### Cases

We conducted a retrospective study of patients newly diagnosed with MPA with renal involvement at the First Affiliated Hospital of Chongqing Medical University from January 2012 to July 2017. All patients met the criteria for the diagnosis of MPA according to Watt’s algorithm [27]. The exclusion criteria were as follows: age younger than 18 years, secondary vasculitis, other primary or secondary glomerular disease (e.g., lupus nephritis, hepatitis B virus or hepatitis C virus infection) and malignancy. Additionally, end-stage renal disease (ESRD) patients at the time of the initial MPA diagnosis were excluded.

### Definition

Renal involvement was defined by one or more of the following criteria: 1) the presence of pauci-immune necrotizing glomerulonephritis in a renal biopsy; 2) the presence of proteinuria≥300 mg/day and haematuria (>5 erythrocytes/high-power field) and/or red blood cell casts, with or without an elevated creatinine level attributed to the disease. The composite of ESRD and doubling of serum creatinine was used as an endpoint of renal death. ESRD was defined as the persistent (more than 3 months) need for renal replacement therapy or permanent reduction in the estimated glomerular filtration rate (eGFR) to <15 ml/min. The doubling of creatinine was defined as the doubling of serum creatinine for more than 3 months. All-cause mortality was defined as death due to any and all causes. Renal survival was defined as independent renal replacement therapy. Comorbidity was assessed by conditions that had been present before the AAV, namely, a history of coronary heart disease, congestive heart failure, peripheral vascular disease, cerebrovascular disease, chronic pulmonary disease, peptic ulcer disease, liver disease or diabetes. Interstitial lung disease (ILD) was defined by the following inclusion criteria: 1) Radiological evidence of ILD on HRCT (such as reticular abnormality or honeycombing with or without traction bronchiectasis), and/or lung function testing consistent with ILD. 2) The exclusion of another possible aetiological factor (such as drugs and dust) in the development of ILD [28].

### Treatment

The immunosuppressive therapeutic regimen was based on corticosteroids and cytotoxic drugs. For induction therapy, oral prednisone was given at a daily starting dosage of 1 mg/kg for 4-6 weeks, with reducing doses over time to 12.5-15 mg by 3 months. Patients with life-threatening or organ-threatening manifestations such as rapidly progressive glomerulonephritis or diffuse alveolar haemorrhage, were given three pulses of intravenous methylprednisolone (7-15 mg/kg/day), followed by daily oral prednisone. Cyclophosphamide (CYC) was given as intravenous pulses (15 mg/kg) every 4 weeks, or it was given orally at a dose of 2 mg/kg/day. For maintenance therapy, daily oral glucocorticoids and/or methotrexate (MTX) (7.5-12.5 mg every week) was given. No patient received rituximab treatment. Thirty-one patients (25.0%) did not receive immunosuppressive treatment, and 5 patients received immunosuppressive treatment 4-12 months after the diagnosis for personal reasons. All survivors were followed up with until July 2018. The average follow-up period was 17 months.

### Data collection

Each patient’s clinical data were collected at the time of diagnosis, such as the age, gender, clinical symptoms and signs at first presentation. The laboratory data were as follows: blood haemoglobin level, serum albumin and creatinine (Scr) level, ANCA-antigen specificity (PR3-ANCA or MPO-ANCA), C-reactive protein (CRP), erythrocyte sedimentation rate (ESR), 24-hour proteinuria and urinalysis. Imaging data, including chest radiograph, computed tomography and magnetic resonance imaging, were collected. Radiological findings were analysed by two observers blinded to the clinical data, with an inter-observer concordance of 95%. The disease activity at diagnosis and organ involvement were assessed according to the BVAS version 3 [29].

### Statistical analysis

Numeric data were presented as the means and standard deviation or medians (interquartile range) and were analysed using Student’s t-test or the Mann-Whitney U test depending on the data distribution. Categorical variables were displayed as frequencies and percentages and were analysed using Chi-squared test and Fisher’s exact test. The Kaplan-Meier method was used to estimate survival, and the log-rank test was used to evaluate differences between groups. Univariate and multivariate Cox proportional hazards regression analyses were performed for prognosis and renal survival. Receiver-operating characteristic curve (ROC) analysis was conducted to determine the variables’ predictive value for prognosis and renal survival. According to the mean values of our patients, we chose 65 years and 30 g/L as cut-off values for the age and serum albumin level, which were also used as cut-off values in previous studies [9, 14, 20, 30]. In China, blood haemoglobin<90 g/L is usually defined as moderate-severe anaemia [31], and the mean blood haemoglobin level of our patients was 88.6 g/L; thus, we used 90 g/L as the cut-off point of blood haemoglobin. Hilhorst et al used 500μmol/L as the cut-off serum creatinine value in prognosis analysis of patients with ANCA-associated glomerulonephritis who presented with a serum creatinine ≥500μmol/L [18], and we also chose 500μmol/L as cut-off point for serum creatinine A P-value of 0.05 or less was considered significant. All the data above were analysed using SPSS 22.0 statistical software (SPSS Inc, Chicago, IL, USA). Power calculation was conducted using the R package ‘powerSurvEpi’ [32, 33].

## Results

### Characteristics of MPA Patients with renal involvement

In total, 124 newly diagnosed MPA patients with renal involvement were enrolled in the study. The characteristics of all patients at diagnosis are described in Table 1. There were 52 men (41.9%) and 72 women (58.1%), ranging in age from 25 and 85 years. Because most of our patients refused to have a kidney biopsy for personal reasons, only 25 patients received a kidney biopsy, and we did not analyse the pathological characteristics of renal lesions.

**Table 1:** Patients characteristics at diagnosis

	MPA (N=124)
Age (years)	63.9±10.6
Gender (male/female)	52/72
MPO-ANCA/PR3-ANCA	117/7
BVAS	15.9±5.7
General manifestation (Myalgia, Arthritis, Fever≥38°C, Weight loss≥2kg), n (%)	68 (54.8%)
Cutaneous involvement, n (%)	8 (6.5%)
Involvement of mucous membranes/eyes, n (%)	2 (1.6%)
Involvement of ear, nose, and throat (ENT), n (%)	1 (0.8%)
Pulmonary involvement, n (%)	76 (61.3%)
Interstitial lung disease, n (%)	59 (47.6%)
Alveolar hemorrhage, n (%)	20 (16.1%)
Cardiovascular involvement, n (%)	15 (12.1%)
Abdominal involvement, n (%)	3 (2.4%)
Nervous system involvement, n (%)	21 (16.9%)
Immunosuppressive treatment, n (%)	93 (75.0%)
Serum creatinine (μmol/L)	353.0 (133.8-582.5)
24h urinary protein (mg/24h)	1608.0 (834.8-2552.2)
Blood hemoglobin (g/L)	88.6±19.6
Serum albumin (g/L)	30.1±6.3
Erythrocyte sedimentation rate (mm/h)	80.1±34.1
Serum C-reactive protein (mg/L)	71.5 (21.7-98.9 )
Coronary heart disease	12 (9.7%)
Heart failure	7 (5.6%)
Cerebrovascular disease	0
Chronic obstructive pulmonary disease	6 (4.8%)
Peripheral vascular disease	0
Liver disease	2 (1.6%)
Peptic ulcer disease	9 (7.3%)
Diabetes	13 (10.5%)

### Risk factors for all-cause mortality in MPA Patients with renal involvement

The overall mortality was 37.1% (46/124) during follow-up. The 6-month overall mortality was 16.1% (20/124), and the 1-year mortality was 21.8% (27/124). The mortality rates were 32.3% (30/93) and 51.6% (16/31) in patients treated with and without immunosuppressive agents, respectively. Furthermore, the 1-year mortality in patients with ILD was 33.9% (20/59) while that in patients without ILD was 10.8% (7/65).

**Fig. 1:**
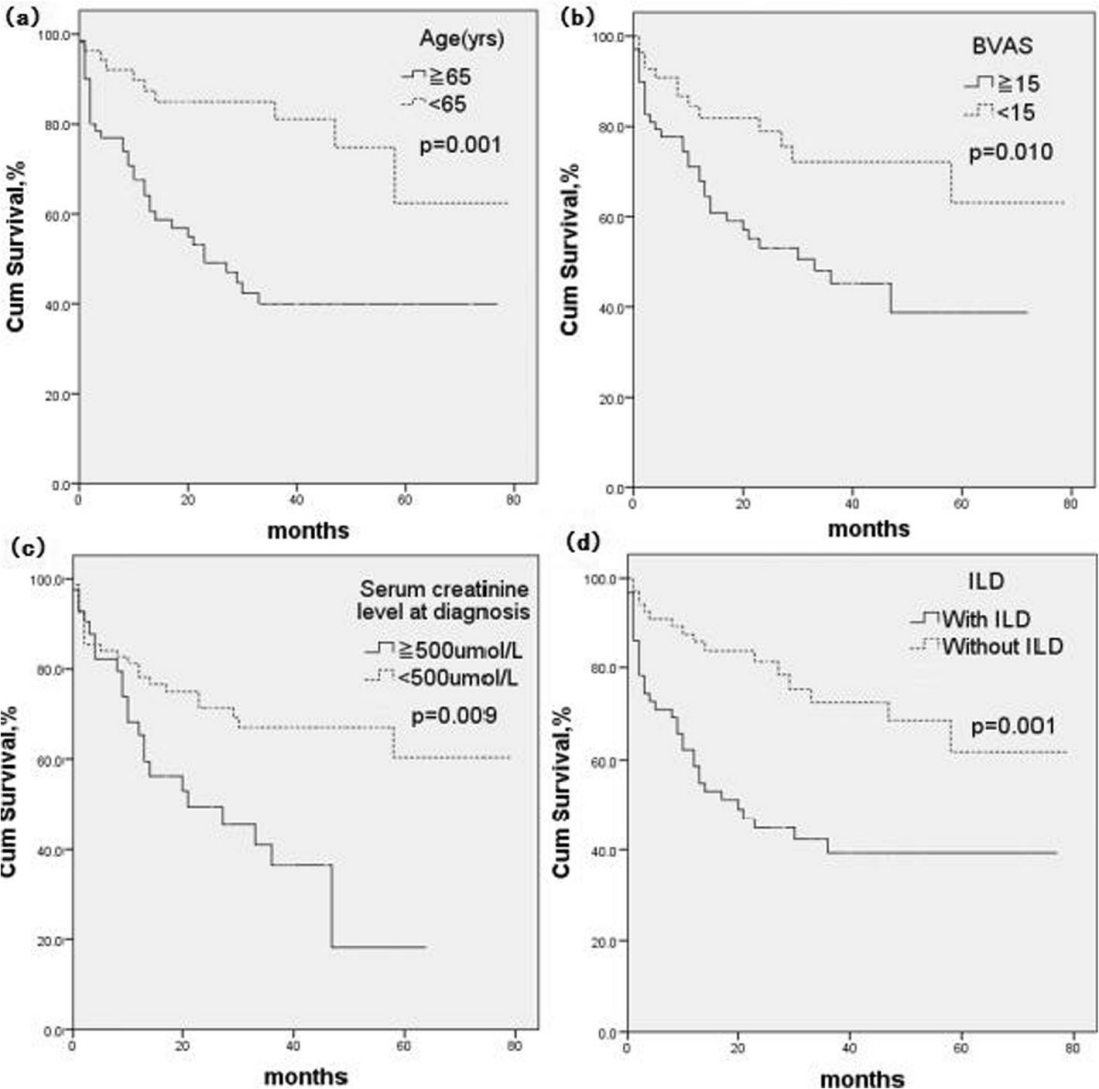
Risk factors for survival of MPA Patient with renal involvement by the Kaplan-Meier survival curve analysis: age (**a**), BVAS (**b**), serum creatinine level at diagnosis (**c**), ILD (**d**)

The Kaplan-Meier survival curve suggested the following parameters were predictors of a poor prognosis: age≥65 years (*P*=0.001), BVAS≥15 (*P*=0.010), serum creatinine at diagnosis≥500 μmol/L (*P*=0.009) and ILD (*P=*0.001) (Figure 1).

Univariate Cox regression analysis showed that age≥65 years, serum creatinine≥500 μmol/L, haemoglobin<90 g/L, BVAS, ILD and abdominal involvement were associated with mortality. Multivariate Cox analysis revealed that age≥65 years (HR: 2.437; *P*=0.021), ILD (HR: 2.366; *P*=0.013) and serum creatinine≥500 μmol/L (HR: 2.207; *P*=0.009) were independent risk factors (Table 2). The power calculation showed that the power of age, ILD and serum creatinine for death was 0.79.

**Table 2:** Univariate and multivariate Cox proportional hazard regression analysis for patient mortality

	Univariate		Multivariate	
	Hazard Ratio(95%CI)	*P* value	Hazard Ratio(95%CI)	*P* value
Male gender	1.594 (0.894-2.843)	1.594		
Age≥65yr (versus <65yr)	3.400 (1.684-6.865)	0.001	2.437 (1.145-5.189)	0.021
Blood hemoglobin <90g/L (versus ≥90g/L)	2.977 (1.544-5.743)	0.001		
Serum creatinine≥500 μmol/L (versus<500μmol/L)	2.172 (1.210-3.898)	0.009	2.207 (1.218-4.000)	0.009
Serum albumin<30g/L (versus ≥30g/L)	1.601 (0.884-2.899)	0.120		
General manifestation (Myalgia, Arthritis, Fever≥38°C, Weight loss≥2kg))	0.711 (0.397-1.273)	0.251		
Cutaneous involvement	0.569 (0.137-2.362)	0.437		
Involvement of mucous membranes/eyes	15.627 (3.307-73.844)	0.001		
Involvement of ear, nose, and throat (ENT)	0.049 (0.001-117546.266)	0.687		
Interstitial lung disease	3.296 (1.750-6.209)	0.001	2.366 (1.199-4.667)	0.013
Alveolar hemorrhage	1.521 (0.754-3.066)	0.241		
Cardiovascular involvement	1.274 (0.569-2.854)	0.556		
Abdominal involvement	4.457 (1.064-18.662)	0.041		
Nervous system involvement	0.708 (0.300-1.672)	0.431		
BVAS	1.114 (1.054-1.177)	<0.001		
Immunosuppressive treatment	0.661 (0.360-1.214)	0.182		
Coronary heart disease	2.164 (1.009-4.643)	0.047		
Heart failure	2.935 (1.241-6.941)	0.014		
Chronic obstructive pulmonary disease	1.466 (0.454-4.736)	0.523		
Liver disease	4.204 (1.006-17.570)	0.049		
Peptic ulcer	1.316 (0.469-3.691)	0.601		
Diabetes	1.522 (0.644-3.598)	0.339		

**Fig. 2:**
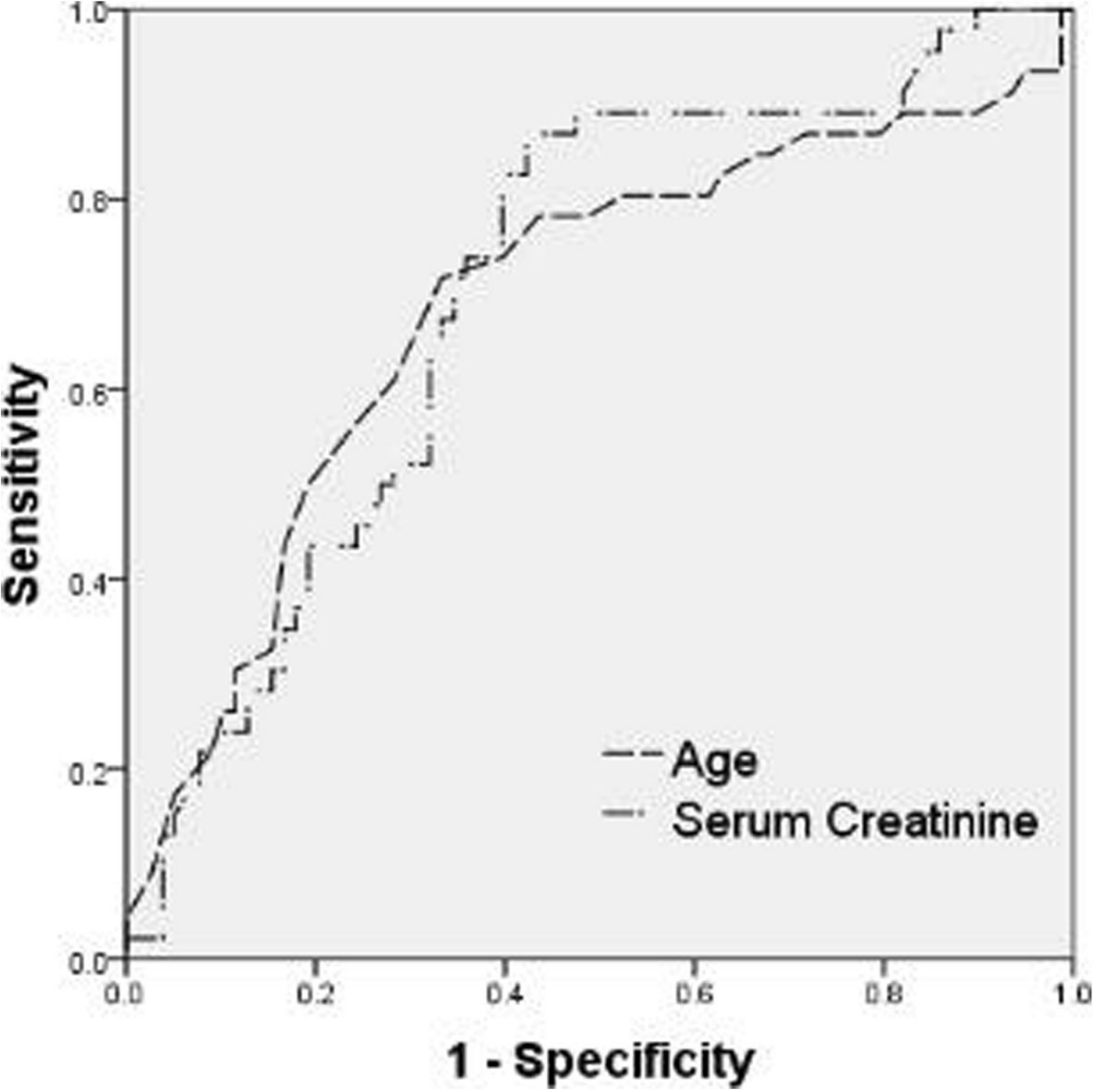
Receiver-operating characteristic curve (ROC) to determine the diagnostic value of age and serum creatinine level at diagnosis for death

Because the age and serum creatinine at diagnosis were found to be associated with death, we performed ROC analysis to assess the predictive value of these variables for death. The area under the ROC curve (AUC) of serum creatinine at diagnosis was 0.705 (95% CI: 0.611-0.799). The optimal cut-off point was 227.0 μmol/L with a sensitivity of 87.0% and a specificity of 57.7%. The AUC of age was 0.690 (95% CI: 0.589-0.792). The cut-off value of age was 66 years, and the sensitivity and specificity were 71.7% and 67.7%, respectively (Figure 2).

### Risk factors for progression to the doubling of serum creatinine or ESRD in MPA Patients with renal involvement

**Fig. 3:**
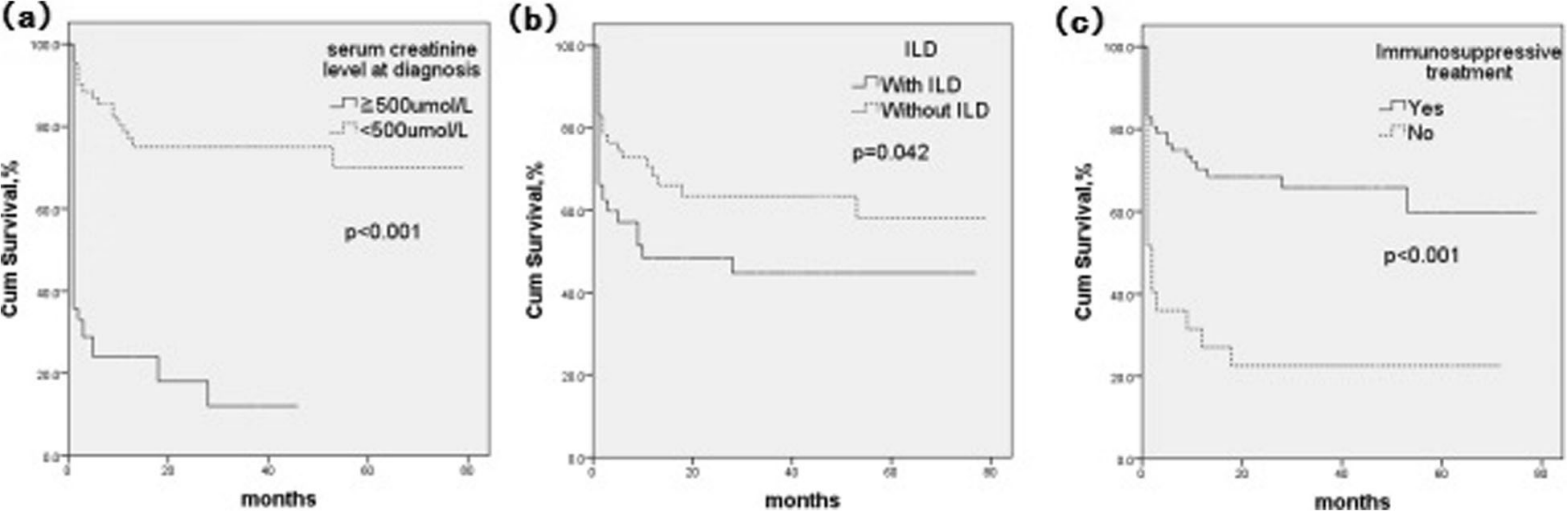
Risk factors for renal survival of MPA patients with renal involvement by the Kaplan-Meier survival curve analysis: serum creatinine level at diagnosis (**a**), ILD (**b**) and immunosuppressive treatment (**c**)

During the follow-up period, 50 patients (40.3%) reached the doubling of serum creatinine or ESRD. Twenty-two (71.0%) of thirty-one patients who did not receive immunosuppressive treatment progressed to the doubling of serum creatinine or ESRD; however, in 93 patients who received immunosuppressive treatment, only 28 (30.1%) progressed to the doubling of serum creatinine or ESRD. Kaplan-Meier survival curve analysis suggested that serum creatinine≥500 μmol/L (*P*<0.001) and ILD (*P*=0.042) were associated with poor renal survival, and patients who received immunosuppressive treatment had a better renal prognosis than those who did not (*P*<0.001) (Figure 3).

Multiple Cox regression analysis confirmed that serum creatinine≥500 μmol/L at diagnosis (HR: 8.236; *P*<0.001) and ILD (HR: 2.649; *P*=0.001) were independent predictors of progression to ESRD, and immunosuppressive treatment (HR: 0.349; *P*=0.001) was a protective factor (Table 3). The power calculation showed that the power of serum creatinine, ILD and immunosuppressive treatment for ESRD and the doubling of serum creatinine was 0.88.

**Table 3:** Univariate and multivariate Cox proportional hazard regression analysis for progression to ESRD

	Univariate		Multivariate	
	Hazard Ratio (95%CI)	*P* value	Hazard Ratio (95%CI)	*P* value
Male gender	1.163 (0.665-2.034)	0.596		
Age65≥yr (versus <65yr)	1.605 (0.899-2.867)	0.110		
Hemoglobin<90g/L (versus ≥90g/L)	2.749 (1.453-5.202)	0.002		
Creatinine≥500μmol/L (versus<500μmol/L)	8.330 (4.515-15.368)	<0.001	8.236 (4.340-15.630)	<0.001
Albumin<30g/L (versus ≥30g/L)	0.832 (0.477-1.450)	0.832		
General manifestation (Myalgia, Arthritis, Fever≥38°C, Weight loss≥2kg)	0.437 (0.247-0.775)	0.005		
Cutaneous involvement	0.042 (0.000-3.749)	0.167		
Involvement of mucous membranes/eyes	2.265 (0.309-16.587)	0.421		
Involvement of ear, nose, and throat (ENT)	2.826 (0.387-20.631)	0.306		
Interstitial lung disease	1.793 (1.020-3.152)	0.042	2.649 (1.461-4.802)	0.001
Alveolar hemorrhage	1.975 (1.030-3.785)	0.040		
Cardiovascular involvement	1.519 (0.713-3.236)	0.279		
Abdominal involvement	1.347 (0.184-9.866)	0.769		
Nervous system involvement	0.470 (0.186-1.185)	0.110		
BVAS	1.062 (1.011-1.114)	0.016		
Immunosuppressive treatment	0.296 (0.168-0.520)	<0.001	0.349 (0.192-0.634)	0.001
Coronary heart disease	2.318 (1.125-4.775)	0.023		
Heart failure	2.448 (0.871-6.878)	0.089		
Chronic obstructive pulmonary disease	0.348 (0.048-2.523)	0.296		
Liver disease	1.116 (0.154-8.095)	0.914		
Peptic ulcer	1.491 (0.590-3.765)	0.398		
Diabetes	1.655 (0.776-3.526)	0.192		

**Fig. 4:**
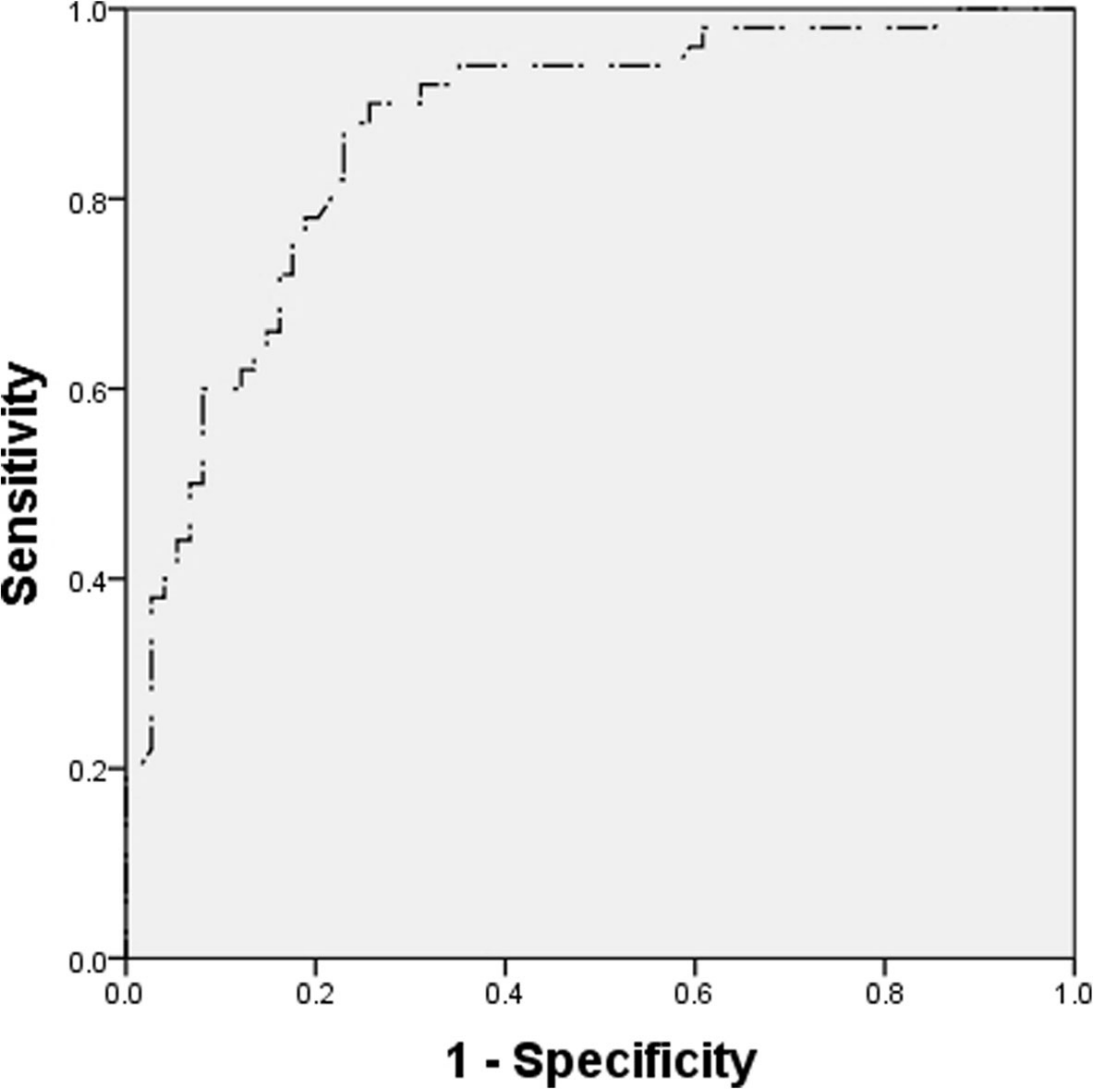
Receiver-operating characteristic curve (ROC) to estimate the diagnostic value of serum creatinine level at diagnosis for renal prognosis

Using ROC, we also confirmed that the serum creatinine level at diagnosis was a predictive factor for renal prognosis. The AUC was 0.870 (95% CI: 0.806-0.934). The optimal cut-off value of serum creatinine at diagnosis was 325.5 μmol/L, and the sensitivity and specificity were 90.0% and 74.3%, respectively (Figure 4).

## Discussion

This study analysed the clinical characteristics and prognostic factors of 124 MPA patients with renal involvement. MPA was previously reported to have a slight male predominance in European countries (male: female ratio varied from 1.08:1 to 1.4:1) [7, 34]. However, in our study, the male-to-female ratio was 0.72:1, which is similar to what has been reported in Japan (0.75:1) [35]. Whether gender affects the incidence of MPA in China remains uncertain, and much more clinical research is needed. Regarding the ANCA phenotype, a study showed that 67% of MPA patients were MPO-ANCA positive in the United Kingdom [7], and a higher ratio of MPO-ANCA positivity was reported in Spain (90.4%) and Japan (varying from 97.1% to 97.4%) [8–10]. We also found a similarly high ratio (94.4%) of MPO-ANCA positivity in MPA patients with renal involvement, which may be attributed to a latitude difference.

In a Japanese study of MPA patients with renal involvement, the survival was 79.5% at 6 months and 71.1% at 12 months [19]. Similarly, in our cohort, the overall survival rate at 6 and 12 months were 83.9% and 78.2%, respectively. Previous studies have shown that age is associated with mortality in AAV patients [6, 12, 13], and we also confirmed age≥65 years was an independent predictor of mortality in MPA patients with renal involvement.

Many studies have reported the association between ILD and MPA [8, 36–38]. AAV patients with ILD have a worse prognosis [36]. In a Japanese cohort study of AAV, 61/156 (39.1%) patients had ILD [8]. Another Japanese AAV study analysed 1,147 AAV patients and found that the 5-year survival rate of AAV patients with ILD was 50.2%, whereas the 5-year survival rate in those without pulmonary involvement was 73.3%, indicating ILD is a predictor of the 5-year mortality [38]. However, the prevalence of ILD was reported to be only 7.2% (14/194) among MPA patients in London, and no difference in survival was noted between patients with and without ILD [23]. In our study, 59 patients (47.6%) had ILD. Additionally, 20/59 (33.9%) of MPA patients with ILD died within the first year, whereas only 10.8% (7/65) of patients without ILD died. Multiple Cox regression showed that MPA patients with ILD had a 2.4-fold increased risk of death compared with those without ILD, suggesting that ILD is an independent predictor of patient survival. This result was further confirmed in Kaplan-Meier survival curve analysis (*P*=0.001). We also found that ILD is an independent predictor of renal survival using multiple Cox regression analysis, and the doubling of serum creatinine or the ESRD rate was 2.6 times higher among MPA patients with ILD than among without ILD. To our knowledge, this is the first report of the association between ILD and ESRD, and the mechanism of ILD associated with the progression of renal dysfunction in MPA patients requires further study.

With the introduction of immunosuppressive treatment, the prognosis of MPA patients was significantly improved with the 1-year survival rate ranging from 56% to 93.9% [24–26]. We found the overall survival was 62.9%, with a 1-year survival of 78.2%. The survival rate of patients with immunosuppressive treatment (67.7%) was higher than it was for those without immunosuppressive treatment (48.4%); however, no significant difference was observed between the groups (p=0.053), and immunosuppressive treatment was also not found to be significantly associated with overall survival using Kaplan-Meier survival and Cox regression analyses, which was likely due to the sample size not being sufficiently large.

Further study showed that the ratio of patients with a serum creatinine level at diagnosis greater than 178 μmol/L was 64.5% (80/124), greater than 443 μmol/L was 37.9% (47/124), reflecting serious renal injury in patients included in our study. Renal involvement is the major clinical feature of MPA. It was reported that mortality in MPA was significantly higher than in GPA and EGPA, mainly because MPA patients developed severe renal failure at the onset of the disease [6, 9]. Wang et al analysed 64 MPA patients with renal involvement showing that the deterioration of renal function is associated with mortality [14], while another report in Japan found that renal function is not associated with overall survival in MPA patients with renal involvement [19]. In this study, a serum creatinine level ≥500 μmol/L at diagnosis was an independent predictive factor for both mortality and ESRD, with hazard ratios of 2.207 and 8.236, respectively. A previous study showed that a peak serum creatinine level greater than 4.5 mg/dl (397.8 μmol/L) at the time of diagnosis is a predictive factor for ESRF or death in AAV patients [39], and another study found that the serum creatinine cut-off level to develop ESRD and require dialysis therapy was 4.6 mg/dl (406.64 μmol/L) in MPA patients with renal involvement [19]. However, in our study, the ROC showed that the serum creatinine cut-off level at diagnosis for a poor prognosis and renal survival were 227.0 μmol/L and 325.5 μmol/L, respectively.

A previous study found that comorbidities were associated with mortality, and this relationship remained independent of other important clinical characteristics [17]. We also analysed the relationship between comorbidities and the prognosis and renal outcome, while comorbidities were not independent predictors of death or ESRD or the doubling of serum creatinine.

Most deaths among AAV patients occurred within the first month after diagnosis because of the high disease activity and side effects of immunosuppressive treatment, such as serious infections and malignancy [11, 25, 40]. A study of 398 Chinese AAV patients also revealed that secondary infection is the leading cause of death (53/153, 39.3%) during the first year after the diagnosis of AAV [6]. In our study, the peak mortality (43.5%) was within the first 6 months after diagnosis. However, because most patients passed away outside of the hospital for unknown reasons, we did not perform analysis on cause of death.

We also found that immunosuppressive treatment is an independent protective factor of renal survival, which is in accordance with the result of a previous study [41]. A delayed diagnosis as well as severe lesions in kidneys may lead to a poor treatment response [6, 9]. These findings suggest that an early diagnosis and timely immunosuppressive treatment are important for the renal recovery of MPA patients and may contribute to a good prognosis, but immunosuppressive treatment should also be used carefully, especially for patients with severe renal dysfunction [6].

Previous studies have proposed disease activity measured by the BVAS as a predictive factor of patient survival in AAV patients with renal involvement [15, 16], while other studies have suggested that BVAS is not statistically correlated with survival in MPA patients with renal involvement [14, 19]. The BVAS was used to evaluate the symptoms of nine systems. In the study, because all the included patients had renal involvement, most of them developed serious renal injury at diagnosis; thus, the median BVAS was high (15.9). Although patients with a higher BVAS score at presentation had a lower survival rate by Kaplan-Meier survival curve analysis, BVAS was not an independent predictor for the prognosis and renal survival by multivariate Cox proportional hazard regression analysis.

This study has some limitations. First, this was a single-centre study, with a sample size that was not sufficiently large, which might constitute a sampling error. Second, most patients passed away outside the hospital for undetermined reasons. Consequently, the cause of death could not be analysed, and other factors (outside of those renal related) that predispose to patient death may not be accounted for. Third, the follow-up period was not long enough because some patients entered the study late, especially those enrolled in 2017, possibly leading to the introduction of bias into prognosis analysis. Multi-centre research that uses large samples and has a longer follow-up duration should be conducted in the future.

## Conclusions

This study showed that age≥65 years, ILD and serum creatinine≥500 μmol/L at diagnosis are independent predictors of mortality in MPA patients with renal involvement. A high level of serum creatinine at diagnosis and ILD are independent predictors of progression to ESRD or the doubling of serum creatinine, while immunosuppressive treatment is a protective factor for renal survival.

## Data Availability

The datasets used and/or analyzed during the current study are available from the corresponding author on reasonable request.

## Abbreviations

AAV: ANCA-associated vasculitis
ANCA: Antineutrophil cytoplasmic antibody
BVAS: Birmingham Vasculitis Activity Score
CRP: C-reactive protein
CYC: Cyclophosphamide
eGFR: Estimated glomerular filtration rate
EGPA: Eosinophilic granulomatosis with polyangiitis
ESR: Erythrocyte sedimentation rate
ESRD: End stage renal disease
GPA: Granulomatosis with polyangiitis
ILD: Interstitial lung disease
MPA: Microscopic polyangiitis
MPO: Myeloperoxidase
MTX: Methotrexate
PR3: Proteinase 3
ROC: receiver operating characteristic curve
Scr: Serum creatinine
